# The dynamics of Ku70/80 and DNA-PKcs at DSBs induced by ionizing radiation is dependent on the complexity of damage

**DOI:** 10.1093/nar/gks879

**Published:** 2012-09-24

**Authors:** Pamela Reynolds, Jennifer A. Anderson, Jane V. Harper, Mark A. Hill, Stanley W. Botchway, Anthony W. Parker, Peter O’Neill

**Affiliations:** ^1^Department of Oncology, Gray Institute for Radiation Oncology & Biology, University of Oxford, Oxford OX3 7DQ and ^2^Rutherford Appleton Laboratory, Central Laser Facility, STFC, Harwell OX11 0QX, UK

## Abstract

DNA double-strand breaks (DSBs) are biologically one of the most important cellular lesions and possess varying degrees of chemical complexity. The notion that the repairability of more chemically complex DSBs is inefficient led to the concept that the extent of DSB complexity underlies the severity of the biological consequences. The repair of DSBs by non-homologous end joining (NHEJ) has been extensively studied but it remains unknown whether more complex DSBs require a different sub-set of NHEJ protein for their repair compared with simple DSBs. To address this, we have induced DSBs in fluorescently tagged mammalian cells (Ku80-EGFP, DNA-PKcs-YFP or XRCC4-GFP, key proteins in NHEJ) using ultra-soft X-rays (USX) or multi-photon near infrared (NIR) laser irradiation. We have shown in real-time that simple DSBs, induced by USX or NIR microbeam irradiation, are repaired rapidly involving Ku70/80 and XRCC4/Ligase IV/XLF. In contrast, DSBs with greater chemical complexity are repaired slowly involving not only Ku70/80 and XRCC4/Ligase IV/XLF but also DNA-PKcs. Ataxia telangiectasia-mutated inhibition only retards repair of the more chemically complex DSBs which require DNA-PKcs. In summary, the repair of DSBs by NHEJ is highly regulated with pathway choice and kinetics of repair dependent on the chemical complexity of the DSB.

## INTRODUCTION

DNA double-strand breaks (DSBs) are biologically one of the most important lesions and may be induced endogenously by reactive oxygen species or exogenously through ionizing radiation and various DNA damaging chemicals. As a result, DSBs produced by these genotoxic agents may possess varying degrees of structural and chemical complexity, and it is the extent of DSB complexity that is thought to underlie the severity of the biological consequences. It is therefore critical that DSBs are repaired correctly to maintain the integrity of the genome and prevent formation of mutations and chromosomal rearrangements or loss, which may ultimately lead to cancer or cell death.

The concept that the ease of repair of DSBs reflects their chemical complexity was proposed based on the observations that a fraction of DSBs induced by sparsely ionizing radiation are very slowly repaired in mammalian cells (1–9) and as a consequence were thought to contribute to the harmful effects of ionizing radiation (1,10,11). While the precise chemical complexity of the different DSB ends was not clearly defined, it was postulated that simple DSBs should be easier to repair than DSBs with more complex structures, for instance when several lesions are proximal to the DSB ends. Insights into the structure and chemical complexity of DSBs (12–15) were first revealed from analysis of the chemical composition of radioactive-iodine-induced DSB ends, which are complex (14). Many of these DSBs possess not only single-stranded overhangs of variable length but also a high frequency of oxidized base modifications and abasic sites directly upstream of the DSB ends. This chemical and structural complexity of DSBs is in addition to the generally formed 3′ blocking ends of DSBs, e.g. 3′-phosphate or 3′-phosphoglycolate moieties (12,14,16–18).

In mammalian cells, DSBs are repaired by two principle pathways, namely non-homologous end joining (NHEJ) and homologous recombination (HR). HR occurs during S or G_2_ phase of the cell cycle and provides greater repair fidelity than NHEJ, which is the major pathway for the repair of DSBs in all phases of the cell cycle (reviewed in (19,20)). Replication-induced DSBs formed at stalled replication forks are normally repaired by HR whereas the majority of DSBs, which are chemically distinct from replication-induced single-ended DSBs, are repaired by NHEJ. NHEJ involves the initial recruitment of Ku70/80 and DNA-PKcs (21–25). Processing of the DSB termini is then thought to occur involving the MRN complex (Mre11, RAD50 and Nbs1), Artemis (2,26,27), PNKP (28,29) and APLF (30). The gaps are subsequently filled by polymerase µ and λ before ligation occurs via XRCC4, Ligase IV and XRCC4 like factor (XLF) (31,32).

Evidence for the inefficient repair of chemically complex DSBs also came from findings using cell lines deficient in either, Artemis (involved in NHEJ) or ATM (Ataxia telangiectasia mutated; involved in DSB signaling and NHEJ), when an increase in the number of persistent DSBs was observed (2,33,34). Confirmation of the inefficient processing of chemically complex DSBs was subsequently confirmed in *in vitro* studies using synthetic oligonucleotide models to simulate chemically complex DSBs with oxidized bases and AP sites at known locations upstream of the DSB ends (5,35). The rate of rejoining of these model complex DSBs by either purified XRCC4/Ligase IV (5) or HeLa cell extracts (35) is indeed severely retarded. Importantly, this retardation seen with HeLa cell extracts could not be explained as a consequence of the 3′-blocking ends of the DSBs (35). Even though the removal of the oxidized bases and AP sites proximal to the DSB termini by base excision repair proteins is inefficient (5), it was inferred that rejoining of these model chemically complex DSBs by cell extracts still occurs prior to removal of the base lesions proximal to the DSB ends (35).

Information is evolving on the structural and chemical complexity of DSBs and on the reduced efficiency of complex DSB processing *in vitro* by NHEJ. To date however, studies on the recruitment of key NHEJ proteins in real-time to sites of DSBs induced in living cells have not considered differential recognition of chemically complex DSBs during NHEJ. These studies have mainly focused on recruitment of NHEJ proteins at early times to DSBs induced by either laser micro-irradiation (24,25,36,37) or irradiation with uranium ions (24), irrespective of consideration of their chemical or structural complexity. The earliest indications that a fraction of DSBs may be repaired by NHEJ in a Ku70/80-dependent DNA-PKcs-independent manner came from Mari *et al*. (25) and Yano *et al*. (36). The few real-time studies at longer times have generally focused on the dependence of the kinase activity of DNA-PKcs on the kinetics of DSB repair (24,38,39). Both ATM phosphorylation and autophosphorylation of DNA-PKcs were found to be essential for efficient DSB repair (40) by facilitating release of DNA-PKcs from DNA ends (38).

Based on the concept that the biological responses to DSBs of different chemical complexity may reflect differential substrate recognition, the aim of the present study was to address whether those NHEJ proteins required for repair of DSBs with greater structural/chemical complexity represent a different sub-set of proteins to those required for repair of less complex (simple) DSBs. Any differences would be indicative of different biochemical processes occurring during NHEJ, of relevance to the potential biological impact of DSBs with differing degrees of complexity. We have therefore used sparsely ionizing ultrasoft X-rays (USX) and multi-photon near infrared laser microbeam (NIR microbeam) as these radiations provide an ideal approach to vary the relative proportions of simple to more chemically complex DSBs induced. USX induce mainly simple DSBs, based on biophysical modeling (41–43) and as inferred from DSB repair kinetics (7,44), in contrast to the induction of a significantly higher proportion of complex DSBs by NIR microbeam irradiation (45–47). The dynamics of the NHEJ proteins involved in the repair of DSBs with differing levels of complexity have been followed in real-time using fluorescently tagged Ku80, DNA-PKcs and XRCC4.

We have shown that Ku70/80 is recruited directly to all DSBs whereas DNA-PKcs is only recruited to longer-lived DSBs, which are suggested to be complex. We also present the first observation in real-time that Ku70/80 is visualized at DSBs induced by sparsely ionizing radiation.

## MATERIALS AND METHODS

### Cell lines and culture conditions

Ku80-EGFP-tagged XR-V15B cells (referred to in the text as Ku80-EGFP cells) were cultured in minimum essential medium (MEM) supplemented with 2 mM L-glutamine. DNA-PKcs-YFP tagged V3 cells (referred to in the text as DNA-PKcs-YFP tagged cells) were cultured in αMEM containing glutamax. All cell culture medium was supplemented with 10% FCS and 100 units/ml penicillin and 100 µg/ml streptomycin in T75 flasks. Cells were maintained at ∼70% confluency at 37°C and 5% CO_2 _humidified air. For USX irradiation, Ku80-EGFP-tagged cells and DNA-PKcs-YFP-tagged cells were plated at 7.5 × 10^4^ cells/dish in 30 mm internal diameter glass walled, 0.9 µm Mylar (polyethylene terephthalate) bottom dishes containing 3 ml of medium and incubated for 48 h at 37°C in 5% CO_2 _humidified air. For all NIR microbeam experiments, cells were plated at 2.0 × 10^5^ cells/dish in 30 mm diameter glass walled, number 1 glass cover-slip bottom dishes containing 3 ml of medium and incubated for 24 h at 37°C in 5% CO_2 _humidified air.

The expression levels of Ku80-EGFP (25) and DNA-PKcs-YFP (24) have been shown to be similar to that of the respective proteins in the wild-type cells. The expression level of XRCC4-GFP is higher than the endogenous levels expressed in wild-type cells (Supplementary Figure S1).

### Real-time irradiations

Cells were cooled before and then maintained at 7°C during irradiation with Al_K_ USX. Cells were irradiated in culture medium at the stated dose (nominal mean dose rate to the cell was ∼2.8 Gy min^−^^1^) through a grid in which gold was deposited in 9 µm wide stripes separated by 1 µm resulting in the cell being irradiated in 1 µm stripes at 10 µm intervals (Supplementary Figure S2 and Supplementary Materials and Methods). Following irradiation, culture medium was replaced with 3 ml of medium warmed to 37°C. Time zero was recorded immediately following addition of warmed medium (37°C) and images were taken at the stated times post-irradiation (at 37°C) using a BioRad Radiance 2000 confocal microscope (Carl Zeiss Ltd., UK) coupled to a Nikon TE2000 microscope (Nikon Instruments Europe B. V., UK).

For NIR microbeam irradiations, cells were incubated with 10 μg/ml Hoechst dye for 10 min prior to irradiation at 37°C and maintained at 37°C throughout the irradiation using the temperature control chamber. The laser was set to a wavelength of 730 nm and a nominal power of 10 mW measured through a ×40 air, numerical aperture 0.95, microscope objective. Cells were irradiated in culture medium using the automated stage to move the cells in a rastering pattern to create damage tracks within the nucleus using a ×60, NA 1.2, water objective to focus the laser microbeam into the cell nucleus. Time zero was recorded immediately following irradiation of the cells (<10 s) and images were collected at the stated times following irradiation using confocal microscopy with a ×60 water objective (EC1, Nikon Instruments Europe B. V., UK) as described above.

Where indicated, 10 µM ATM kinase inhibitor (Merck Chemicals, UK) or 250 nM PARP inhibitor (Kudos, UK) was added 45 min prior to damage induction. The inhibitor concentrations were chosen based on either the IC_50_ or EC_50_ and have been recommended by the manufacturer for inhibition of DSB repair, while having low levels of cytotoxicity in the absence of DSBs. ATM kinase inhibitor has an IC_50_ of 13 nM with little cross reactivity ≤10 µM) (48) and Kudos PARP inhibitor an IC_50 _of 5 nM (49,50). To inhibit histone deacetylation (HDAC) either 5 mM of sodium butyrate (Sigma Aldrich, UK) or 1.3 µM of trichostatin A (TSA) was added 16 h prior to irradiation (51).

### Quantification of protein intensity from real-time confocal images

The confocal microscope images of recruitment of proteins in real-time were analysed by measuring the intensity of the fluorescently tagged protein of interest using Quantity One® software. In the real-time experiments, the fluorescence intensity of the protein along an irradiation track within an individual cell over the repair time course was determined by analysing a minimum of 10 cells per experiment. The foci track was selected at each time point post-irradiation and the nuclear background intensity was determined by selecting an un-irradiated area within the cell nucleus. The intensity of the protein was calculated by subtracting this nuclear background from the intensity of the foci track. The average intensity of all of the foci tracks was calculated and all intensities at different times post-irradiation were normalized to the maximum fluorescence intensity determined at earlier times (maximum relative fluorescence of 1). A minimum of three experiments were carried out and the data are expressed as the mean together with the SEM.

### Kinetic analysis of loss of fluorescence intensity of the tagged proteins from sites of damage

The rate of loss of fluorescence intensity for Ku80-EGFP and DNA-PKcs-YFP from DSBs induced by either NIR microbeam or USX irradiation was analysed using Origin software® assuming either mono-exponential kinetics ((equation (1))
(1)
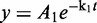

or bi-exponential kinetics (equation (2))
(2)
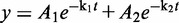

where *y* is the relative fluorescence intensity of the fluorescently tagged protein at the damage sites at time *t*, k_1_ and k_2_ are the first-order rate constants for loss of fluorescence intensity by reactions 1 or 2, *A*_1_ and *A*_2_ are the initial levels of the fluorescently tagged protein which are involved in the DSB repair processes with rate constants k_1_ and k_2_. The fraction of the fluorescently tagged protein associated with the damage site decaying with rate constant k_1_ (reaction 1) is given by
(3)
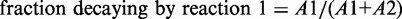

and rate constant k_2_ (reaction 2) is given by
(4)
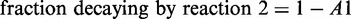



The half-life (*t*_½_) for each reaction is given by
(5)
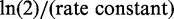



**Figure 1.**
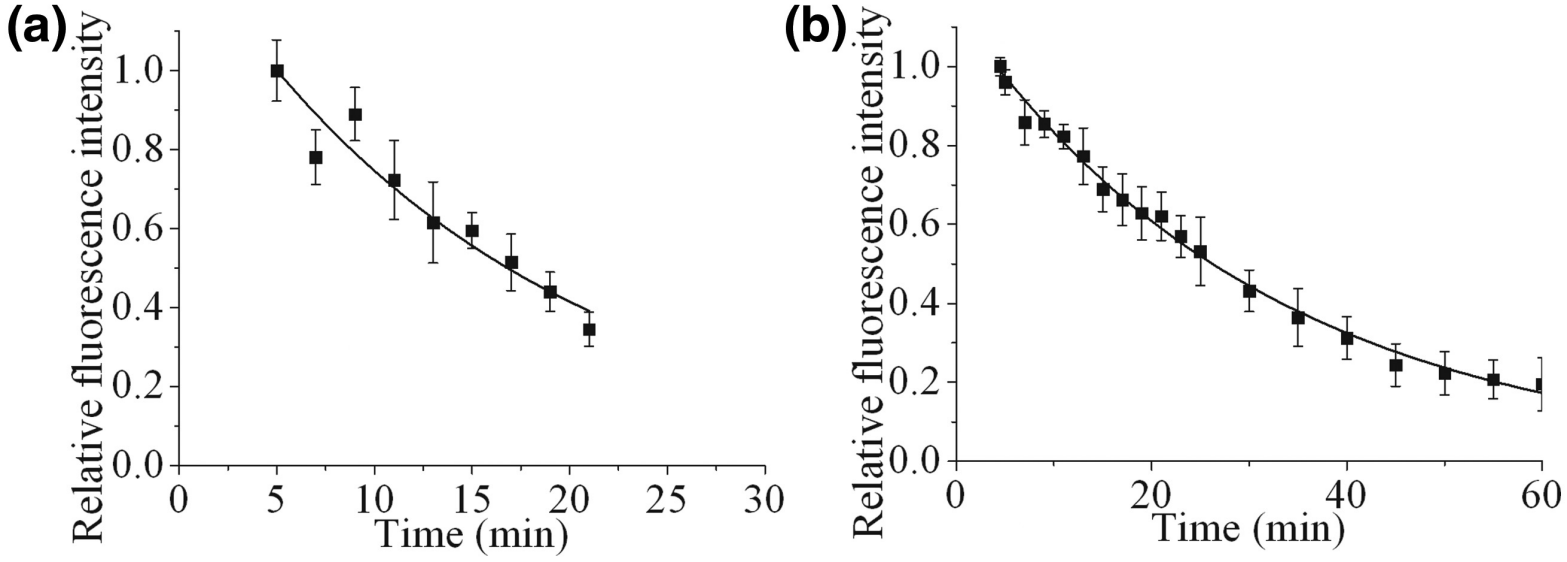
Dependence of recruitment and loss of fluorescence intensity of Ku80-EGFP on time following USX irradiation of Ku80-EGFP tagged cells at 7°C with a dose of (**a**) 27 Gy or (**b**) 137 Gy followed by incubation at 37°C. For real-time analysis, each point represents the relative fluorescence intensity normalized to the intensity at ‘zero time’ following irradiation and maintaining the cells at 37°C. The kinetic analysis to obtain the best fit to the experimental data are shown as solid lines and described in the Materials and Methods section.

**Figure 2.**
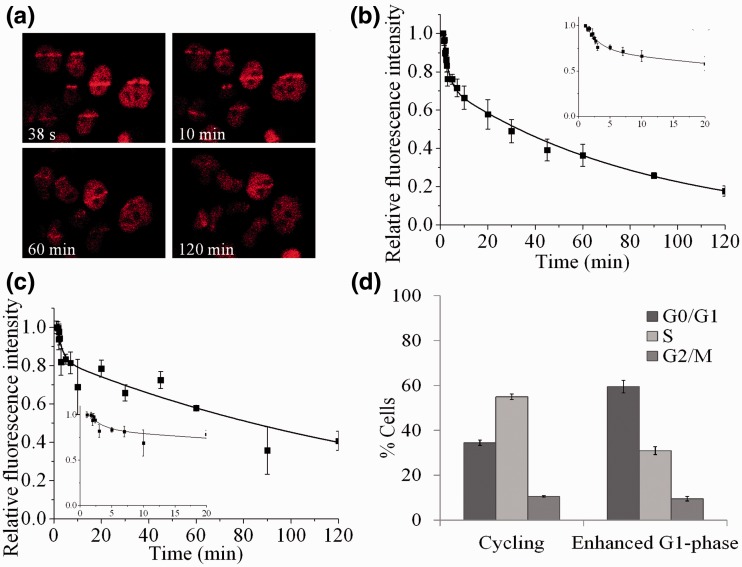
Dependence of the recruitment and loss of fluorescence intensity of Ku80-EGFP in (**a**) and (**b**) exponentially growing and (**c**) serum-starved cells on time following NIR microbeam irradiation with 730 nm photons (at a power of 10 mW through a ×60 objective). For real-time analysis, each point represents the relative fluorescence intensity normalized to the intensity at ‘zero time’ following irradiation and maintaining the cells at 37°C. The kinetic analysis to obtain the best fit to the experimental data are shown as solid lines and described in the Materials and Methods section. The insets represent the data expanded to show the first 20 min in more detail. (**d**) Flow cytometry analysis to show the cell cycle distribution of exponentially growing and serum-starved cells.

**Figure 3.**
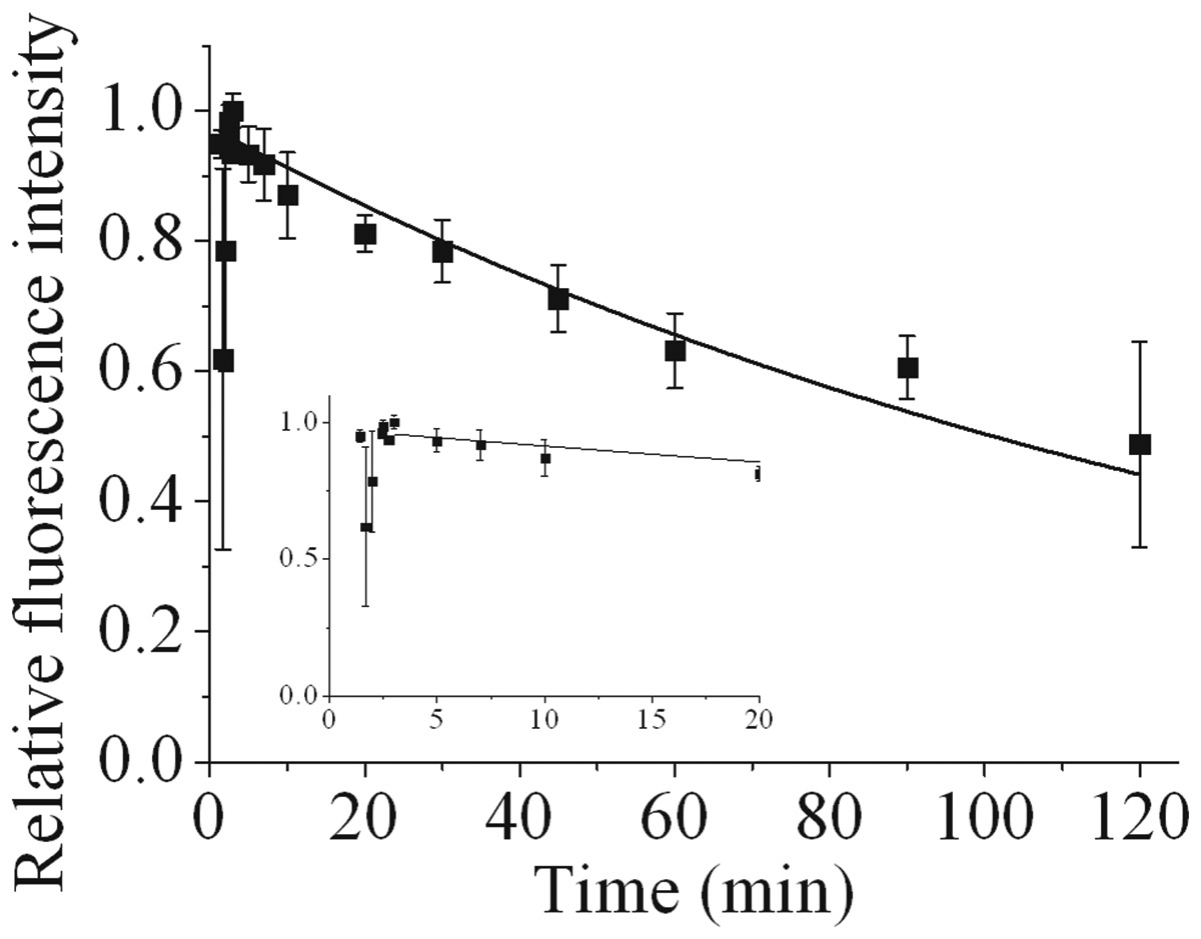
Dependence of the recruitment and loss of fluorescence intensity of DNA-PKcs-YFP on time following NIR microbeam irradiation with 730 nm photons (at a power of 10 mW through a ×60 objective) in cycling cells. For real-time analysis, each point represents the relative fluorescence intensity normalized to the intensity at ‘zero time’ following irradiation and maintaining the cells at 37°C. The kinetic analysis to obtain the best fit to the experimental data are shown as solid lines and described in the Materials and Methods section. The insets represent the data expanded to show the first 20 min in more detail.

The data points in figures (Figures 1, 2b, c, 3, 5 and 6, and Supplementary Figures S3b, S4, S6 and S7) were fitted using either equation (1) or (2) to obtain the half-lives for the different reactions and the proportion of the loss of fluorescence in the case of bi-exponential fits to the two first-order components. The best fit curves to the experimental data are shown as solid or dashed lines.

## RESULTS

### Ku70/80 is recruited to USX and NIR microbeam-induced DNA DSBs

With the exception of the studies by Mari *et al*. (25) and Kim *et al*. (37) recruitment of Ku70/80 to DSBs in real-time have generally been difficult to observe as only a few molecules are recruited to damage sites against the high nuclear levels, estimated to be ∼400 000 molecules per nucleus (52–54). Using USX, which are highly attenuated, in conjunction with a patterned 1 µm thick gold shield (55) (Supplementary Figure S2), distinct tracks of DSBs are induced, visualized as phosphorylated H2AX foci (γH2AX). These observations of γH2AX foci tracks verify that DSBs are induced using the USX set-up described. In similar experiments, the recruitment of Ku80-EGFP to DSB tracks was seen within 5 min, the earliest time point recorded, following irradiation with a dose of 27 Gy of USX (Figure 1a). Due to the low dose rate of the USX set-up, the irradiations were performed at 7°C to minimize repair during the irradiation period. As the cells were subsequently warmed to 37°C, the recruitment and repair times of DSB will also reflect the time required to attain 37°C (Supplementary Figure S3a). The time-dependent loss of fluorescence intensity of Ku80-EGFP in the DNA damage tracks proceeds via an exponential decay with a calculated half-life life (*t*_½_) of 12 ± 5 min. To increase sensitivity, particularly at longer times, the USX dose was increased to 137 Gy (Figure 1b). The *t*_½_ for exponential loss of the majority of the fluorescence intensity of Ku80-EGFP is 19 ± 4 min, suggesting Ku80-EGFP is involved during the repair of the majority of DSBs. However it is now apparent at the higher dose that ∼15% of the total fluorescent intensity persists, indicative of the presence of a small percentage of longer-lived DSBs, which also utilize Ku80 during their repair.

From pulse field gel electrophoresis (PFGE) DSB repair data, it had previously been suggested that the majority of USX-induced DSBs are simple (7,44,56). The USX findings presented here on the dynamics of loss of Ku80-EGFP at DSBs are consistent with the formation and repair of these simple DSBs. To explore this further, the kinetics of loss of Ku80-EGFP from DSBs were determined at 37°C following NIR microbeam irradiation (Figure 2a) (45), which produces a greater fraction of complex DSBs. Ku80-EGFP is rapidly recruited to NIR microbeam-induced DSBs, with maximal relative fluorescence intensity seen within 1 min following irradiation, consistent with the findings of Mari *et al*. (25) (Figure 2a). Within the first 10–15 min, a rapid loss of ∼30% of Ku80-EGFP relative fluorescence occurs, followed by a slower loss during the repair of the remaining DSBs (Figure 2a and quantified in Figure 2b). These observations are consistent with the prediction that a greater fraction of complex DSBs are produced by NIR microbeam irradiation (45). We questioned whether the initial rapid loss of Ku80-EGFP seen with NIR microbeam irradiation is cell-cycle dependent. The initial loss of ∼30% of the Ku80-EGFP fluorescence is independent of the percentage of cells in a given phase of the cell cycle as seen by the similar kinetics in cycling (Figure 2b) and enhanced G_1_-phase cells (Figure 2c and d). The loss of Ku80-EGFP fluorescence in exponentially growing and enhanced G_1_-phase cells occurs via bi-phasic kinetics. The fast component observed decays with a *t*_½_ of 1.5 ± 1 min (34 ± 10% of the DSBs) which is independent of the phase of the cell cycle. The slower component of loss occurs with *t*_½_ of 72 ± 35 min (66 ± 10% of DSBs) and 120 ± 32 min (59 ± 5% of DSBs) for exponentially growing and enhanced G_1_-phase cells, respectively.

The higher value of *t*_½ _for the fast component following USX irradiation compared with NIR microbeam irradiation emphasizes the differences in the temperature during irradiation (7°C versus 37°C, respectively) and the time to reach 37°C following USX irradiation (Supplementary Figures S3a and b). The differences in the proportion of fast to slow components for loss of fluorescence intensity of Ku80-EGFP during repair of DSBs induced by USX and NIR microbeam irradiation is consistent with a greater fraction of complex DSBs induced by NIR microbeam irradiation. Additionally, the rapid loss of Ku80-EGFP relative fluorescence during repair of DSBs represents processing by NHEJ and not HR, since Ku80-EGFP loss occurs before the time for maximal recruitment of RAD51 (a key protein in HR) and loss of γH2AX following NIR microbeam irradiation (45).

In addition to the classical NHEJ pathway, cells may also use a Ku-independent back-up NHEJ (B-NHEJ) pathway which involves poly(ADP)ribose polymerase (PARP1) and ligase III (57,58). To verify that the B-NHEJ pathway does not play a role in the repair of DSBs in Ku70/80 proficient cells (58,59), real-time recruitment and loss of Ku80-EGFP was visualized in the presence and absence of a PARP1 inhibitor. The recruitment and loss of Ku80-EGFP at DSBs induced by either NIR microbeam (Supplementary Figure S4a) or USX-radiation (Supplementary Figure S4b) is unaffected when PARP1 activity is inhibited. Therefore, we have verified that B-NHEJ does not appear to have a substantial role in DSB repair in Ku70/80 proficient cells.

### Repair of DSBs involving DNA-PKcs induced by NIR microbeam-irradiation proceeds predominantly via a single repair process with slow kinetics

Following NIR microbeam irradiation of DNA-PKcs-YFP-tagged cells, DNA-PKcs-YFP is recruited to NIR microbeam-induced DSBs within 1 min following irradiation, reaching maximal relative fluorescence intensity within 3 min, consistent with the observations by Uematsu *et al*. (24) (Figure 3). In contrast to the observations with Ku80-EGFP, a significant rapid loss of fluorescence of DNA-PKcs-YFP was not seen, suggesting that DNA-PKcs is not directly involved during the fast component of repair of a sub-set of DSBs. The first order loss of DNA-PKcs-YFP fluorescence intensity (*t*_½_ of 78 ± 50 min) is consistent with the slow component seen during DSB repair in cycling Ku80-EGFP cells (Figure 2b). It is therefore concluded that DNA-PKcs is mainly recruited to the slowly repairing DSBs which also require Ku80.

**Figure 4.**
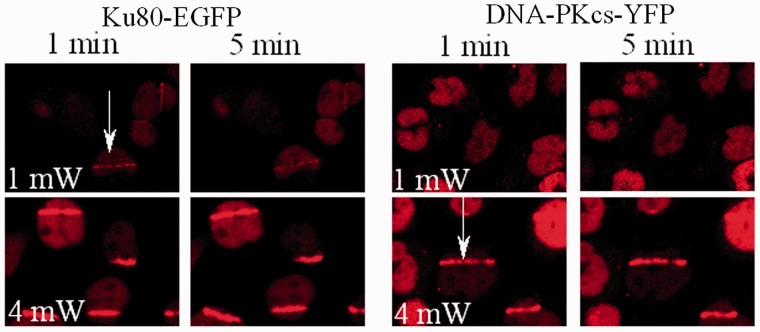
Power dependency of the intensity of fluorescence of Ku80-EGFP and DNA-PKcs-YFP at DSB induced in the, respectively, tagged cells following NIR microbeam irradiation with 730 nm photons (using a ×60 water microscope objective) at 37°C. Real-time confocal microscope images of the respective tagged proteins following NIR microbeam irradiation were taken at 1 min and 5 min following irradiation.

Biochemical evidence has shown that 1–2 molecules of Ku80 and DNA-PKcs bind to their respective DSB substrate ends (38,60,61). As such, if DNA-PKcs is recruited mainly to the slowly repairing sub-set of DSBs, whereas Ku80 is recruited to all DSBs, then reducing the laser power and hence the overall yield of DSBs should result in observation of Ku80-EGFP at DSB sites at lower laser powers than that required to see DNA-PKcs-YFP. This prediction was verified from a NIR microbeam power dependency, as Ku80-EGFP recruitment was seen at DSB sites at 1 mW, whereas recruitment of DNA-PKcs was visualized only at ∼4 mW (Figure 4). Similarly, recruitment of Ku80-EGFP can be seen at lower doses of USX down to ∼6 Gy compared with the higher doses required to see recruitment of DNA-PKcs-YFP (54 Gy) (Supplementary Figure S5a). Both these observations are consistent with the prediction that fewer complex DSBs are induced by USX than by NIR microbeam irradiation (45). To verify that these observed differences are not attributed to differences in repairability of DSBs in the different cell lines, the induction and repair of DSBs (as represented by γH2AX foci) was found to be similar in both Ku80-EGFP tagged XRV15B cells and DNA-PKcs-YFP tagged V3 cells, when irradiated with 1 Gy of γ-radiation (Supplementary Figure S5b).

### XRCC4 is recruited to USX and NIR-microbeam-induced DNA DSBs

**Figure 5.**
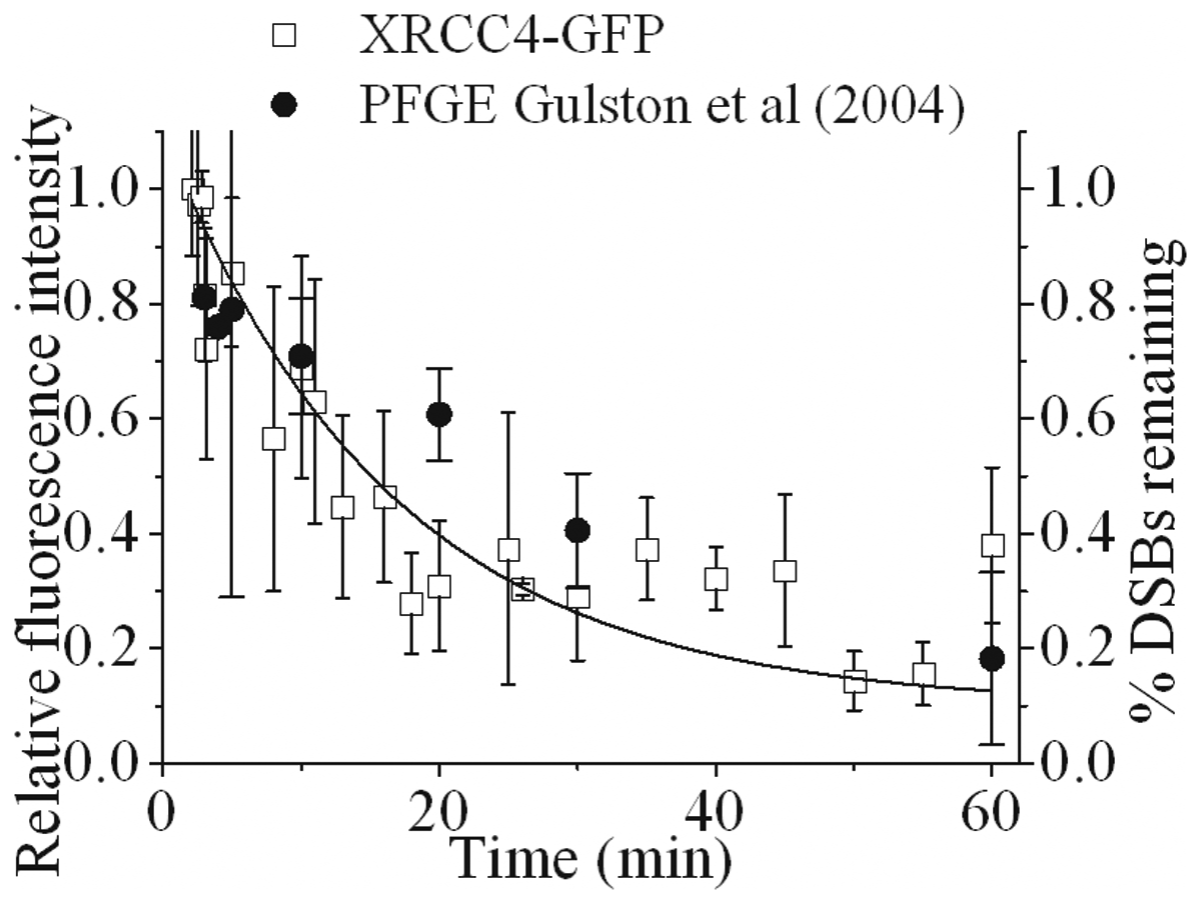
Time course of the repair of DSBs visualized by real-time recruitment and loss of fluorescence intensity of XRCC4-GFP following NIR microbeam irradiation with 730 nm photons (at a power of 10 mW through a ×60 objective) in cycling XRCC4-GFP tagged XR1 cells and PFGE data taken from Gulston *et al.* (56). The kinetic analysis to obtain the best fit to the experimental data are shown as solid lines and described in the Materials and Methods section.

Since Ku80 is lost from rapidly repairing simple DSBs, the dynamics of recruitment and loss of fluorescence intensity of XRCC4-GFP at NIR-microbeam-induced DSBs in XR1 (XRCC4 deficient) cells complemented with XRCC4-GFP (Supplementary Figure S1) was determined, due to its role in the final step of DSB ligation. XRCC4-GFP is recruited to NIR microbeam-induced DSBs within 1 min followed by a loss of fluorescence intensity (*t*_½_ of 8 ± 1 min) (Figure 5) at a rate slightly slower than that seen for the fast component of loss of fluorescence intensity of Ku80-EGFP at DSB (*t*_½_ of 1.5 ± 1 min). From these differences it is suggested that Ku80 dissociates from rapidly repairing DSBs shortly before their ligation. This rate of loss of fluorescence intensity of XRCC4-GFP at the rapidly repairing DSB (Figure 5) is comparable with the rate of repair of the majority of γ-radiation-induced DSBs determined by PFGE (56). In contrast, loss of XRCC4-GFP was not seen from the slower repairing DSBs as would be predicted, since the ligation step involving XRCC4 would not now be rate determining. The similarity between the kinetic data (Figure 5) also confirms that the increased expression level of XRCC4-GFP compared to the wild-type levels does not affect the rate of repair of DSBs (Figure 5 and Supplementary Figure S1).

### The inhibition of ATM activity retards the repair of a sub-set of DSBs that utilize both Ku80-EGFP and DNA-PKcs-YFP

**Figure 6.**
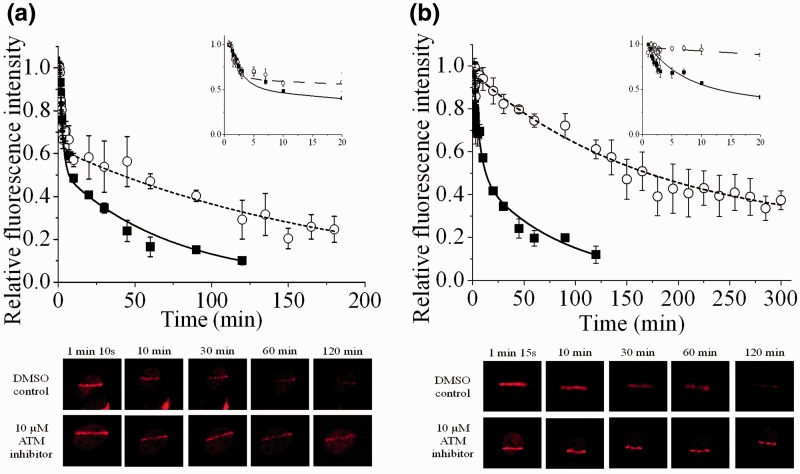
Effects of 10 µM ATM kinase inhibitor on the real-time recruitment and loss of fluorescence intensity of (**a**) Ku80-EGFP and (**b**) DNA-PKcs-YFP following NIR microbeam irradiation of Ku80-EGFP and DNA-PKcs-YFP tagged cells, respectively, with 730 nm photons (at a power of 10 mW using ×60 objective) and at 37°C. The kinetics of loss of fluorescence intensity of the respective proteins in Dimethylsulfoxide (DMSO)-treated control cells (black boxes) and cells treated with ATM inhibitor (open circles) were analysed and represent the mean of three independent experiments ± SEM with the solid (control) and dotted (inhibitor) lines showing the fit of the exponential decays to the data points. The insets represent the data expanded to show the first 10 min in more detail. The images are representative of the fluorescence level over the repair time course in control and ATM inhibitor treated cells.

ATM has been shown to be involved in phosphorylation of DNA-PKcs at Thr-2609 cluster, facilitating its release from DSB ends (24,40). We therefore investigated the effects of inhibition of ATM activity on the kinetics of loss of fluorescence intensity of Ku80-EGFP and DNA-PKcs-YFP during the repair of DSBs of varying complexity. The inhibition of ATM does not alter the expression levels of Ku80-EGFP/DNA-PKcs-YFP or the actual fluorescence intensity of either Ku80-EGFP or DNA-PKcs-YFP at early times when recruited to the NIR microbeam-induced damage sites (Figure 6 images).

The recruitment of Ku80-EGFP to NIR microbeam-induced DSBs is unaffected by inhibition of ATM, with maximum levels of relative fluorescence observed within 1 min in control and ATM-inhibited cells (Figure 6a inset and images). Additionally, the initial rapid loss of Ku80-EGFP relative fluorescence over the initial 10 min is not affected by the presence of the ATM inhibitor (Figure 6a). In contrast, the subsequent slower loss is retarded by the ATM inhibitor (Figure 6a) by a factor of ∼3.5, when the *t*_½_ value increases from 30 ± 14 min to 107 ± 18 min in the presence of the inhibitor. It is suggested that ATM is mainly involved in NHEJ repair of the slower repairing DSBs.

To see if the rate of loss of Ku80-EGFP during repair of DSBs induced by USX in the presence and absence of the ATM inhibitor mainly effects the slower repairing DSBs, the cells were irradiated at 37°C (radiation time ∼12 min). Ku80-EGFP is recruited rapidly during the USX-irradiation period in the presence and absence of the ATM inhibitor. Although difficult to quantify since a fraction of the DSBs will have been repaired during the irradiation period at 37°C, it is apparent that the rate of loss of relative fluorescence of Ku80-EGFP is slower in the presence of the ATM inhibitor particularly at longer times (Supplementary Figure S6). The effect of ATM can be seen from the difference in the level of fluorescence, relative to the intensity at zero time, of 45% in the presence of the ATM inhibitor compared with only 20% in control cells 35 min post-irradiation. The inhibitory effect by the ATM inhibitor on slower repairing DSBs induced by USX is consistent with that seen following NIR microbeam radiation.

Having shown that the ATM inhibitor mainly affects the slower repairing DSBs, we then tested if the ATM inhibitor would retard the loss of DNA-PKcs-YFP, which is recruited mainly to the slower repairing DSBs. The recruitment of DNA-PKcs-YFP to NIR microbeam-induced DSBs is unaffected by the inhibition of ATM, with similar maximum levels of relative fluorescence observed within 1 min in control and ATM-inhibited cells (Figure 6b inset and images). However the loss of the majority of the DNA-PKcs-YFP relative fluorescence is retarded by the ATM inhibitor (Figure 6b) by a factor of ∼3.3. The *t*_½ _value increases from 42 ± 11 min to 138 ± 59 min in the presence of the inhibitor. Taken together with the finding for Ku80, it is confirmed that ATM inhibition affects mainly the slower repairing DSBs through retention of not only DNA-PKcs but also Ku80 at complex DSB ends.

## DISCUSSION

The notion that the repairability of more chemically complex DSBs is less efficient, has led to the concept that the extent of DSB complexity underlies the severity of the biological consequences (1–3,9,19). Differences in the fraction of DSBs of varying chemical complexity induced by USX (7) or NIR microbeam radiation (45) used here confirmed our findings from real-time recruitment and loss of NHEJ proteins to DSBs. It is proposed that at least two distinct sub-pathways of NHEJ are utilized in the repair of the different types of DSBs, distinguished through their complexity. The fast component of DSB repair is Ku70/80 dependent but DNA-PKcs independent, whereas the slower component of DSB repair is dependent on both Ku70/80 and DNA-PKcs. The latter component also involves ATM, even though Ku80 (25), DNA-PKcs (24), XRCC4 and XLF (36) are all recruited rapidly to the induced DSBs. It is proposed that the fast component of DSB repair represents predominantly simple DSBs whereas the slower component represents DSBs with more complex structures, for instance when several lesions are proximal to the DSB ends. This proposal is consistent with the observation that increasing the proportion of complex relative to simple DSBs using NIR microbeam radiation (45) results in rapid loss of Ku80 from a smaller proportion of DSBs (Figure 2b) than seen with USX (Figure 1a), which induces mainly simple DSBs (7,44). Additionally, we have shown from the real-time kinetics for recruitment and loss of Ku70/80, that the choice of the NHEJ repair pathway for these different types of DSBs is independent of the phase of the cell cycle. Our study clearly shows that DSBs of different chemical complexity should be considered as different substrates with regard to the NHEJ proteins required for their repair. In the majority of real-time studies to date, it has not been considered generally that repair of laser-induced DSBs may be dependent on their complexity (23–25,36).

We present the first observation of recruitment of NHEJ proteins to sparsely ionizing radiation-induced DSBs in real-time, seen from the accumulation of Ku80 as distinct foci tracks to USX-induced DSBs. The subsequent rate of repair of the majority of the DSBs (∼85–90%) is similar to that determined from PFGE for repair of DSBs induced by USX (7,44,56). In contrast to the rapid loss of Ku80, DNA-PKcs is not directly involved in the fast component of DSB repair when induced by either USX or NIR microbeam radiation. DiBiase *et al*. (62) showed that the kinetics of DSB repair in MO59J and MO59K cells are the same, although the proportion repairing by fast and slow components shifts toward the slow component with mutated DNA-PKcs. However, this shift was not verified using the same cell lines when using γH2AX as the marker for DSBs (63).

The loss of XRCC4, a monitor of DSB ligation, from the DSBs induced by NIR microbeam occurs with a similar rate (*t*_½_ of 8 min) to that for loss of the majority of DSBs induced by γ-radiation at 37°C as determined by PFGE (44,56) (Figure 5). The rejoining of DSBs with a *t*_1/2_ of 8 min implies that 3′ phosphates and/or 3′ phosphoglycolates are removed quickly from the DNA ends prior to ligation. From the dynamics of Ku80 recruitment to and loss from simple DSBs, it is inferred that XRCC4 is recruited to DSBs prior to Ku80 release, which occurs shortly before ligation of DSBs, in a DNA-PKcs independent manner. Previous investigations using DNA-PKcs null cells have indirectly shown that Ku70/80 and XRCC4 and XLF are able to repair NIR microbeam-induced DSBs, suggesting that activity of the XRCC4/ligase IV/XLF complex is the switch from processing to repair/ligation (25,36).

The repair of complex DSBs occurs by a single process at a rate which is at least 10x slower than that for the loss of Ku80 from simple DSBs in a Ku70/80 and DNA-PKcs-dependent manner. This slower repair of complex DSBs probably reflects the additional processing necessary to remove lesions close to the DSB ends (1,4,5,10,11,13,35). Previously, Riballo *et al*. (2) suggested from indirect evidence using a variety of DSB repair deficient cell lines that the repairability of DSBs, determined using PFGE, involves ATM and DNA-PKcs but only for a sub-set of DSBs. Additional confirmation for these two distinct NHEJ sub-pathways in the repair of simple and complex DSBs comes from differences in the dose/power responses for accumulation of Ku80 relative to that of DNA-PKcs at USX and NIR microbeam-induced DSBs, even when taking into account differences in the excitation coefficients between YFP and GFP at 488 nm. Moreover, the inability to visualize DNA-PKcs-YFP at low NIR microbeam powers and USX doses, when Ku80 is visualized, is not due to differences in the number of molecules recruited to the DSB termini. It has previously been shown that 1–2 molecules of either Ku70/80 or DNA-PKcs bind to their respective DSB substrate ends (38,60). Additionally, >2 Ku-heterodimers bound to DSB ends have been shown to inhibit ligation (60).

Although inhibition of ATM kinase activity does not affect the recruitment of either Ku80 or DNA-PKcs to NIR microbeam-induced DSBs, the inhibitor mainly retards the processing of the slower repairing DSBs, namely the complex DSBs, by a factor of ∼3 (Figure 6). ATM has been shown to be involved in phosphorylation of DNA-PKcs at Thr-2609 in a DSB-dependent manner resulting in its release from DSB ends (40). The persistence of DNA-PKcs at DSB sites depends on the phosphorylation/autophosphorylation status of DNA-PKcs, since Uematsu *et al*. (24) showed that the rate of release of DNA-PKcs from DSBs is also retarded when the autophosphorylation sites are mutated. Phosphorylation of DNA-PKcs induces a conformational change, facilitating the release of DNA-PKcs from DNA ends (38). Our findings are consistent with retardation of phosphorylation of DNA-PKcs by ATM resulting in ‘slowing down’ the release of not only DNA-PKcs but also Ku80 from mainly complex DSBs. The involvement of ATM in the repair of complex DSBs is consistent with our previous studies showing that ATM persists at NIR microbeam-induced damage for up to 6 h post-irradiation in the absence of an ATM inhibitor (45). It is therefore proposed that phosphorylation of the DNA-PK complex, known to cause a conformation change (38), potentially allows Ku80 to be released from the DNA ends together with DNA-PKcs. In contrast, ATM inhibitors do not affect the loss of Ku80 from DNA ends during the repair of simple DSBs when DNA-PKcs is not directly required. It is suggested that shortly prior to the release of DNA-PKcs and Ku80, the XRCC4/ligase IV complex is recruited to facilitate ligation.

Chen *et al*. (40) indicated that phosphorylation of DNA-PKcs at the Thr-2609 site may be important for the activation of the endonuclease activity of Artemis by creating a docking site, consistent with the notion that complex DSBs require DNA end processing prior to ligation. In accordance with this data ATM and Artemis have previously been implicated in the repair of complex DSBs (2,64–66). An alternative view is that this sub-set of slowly repairing DSBs when formed in heterochromatin requires ATM to relax the heterochromatin before repair may be initiated (67,68). If this were the case in the cell lines used in our study, we would have predicted that the fractions of slow relative to rapidly repairing DSBs induced by USX or NIR microbeam and utilizing NHEJ should have been very similar. Additionally we would have predicted that the recruitment of both Ku80 and DNA-PKcs, which occurs within seconds to the tracks of radiation-induced DSBs, would be impeded if inhibition of the kinase activity of ATM significantly impaired the recruitment of proteins to heterochromatin-associated DSBs. Previous studies at the DNA damage level have inferred that the efficiency of rejoining of radiation-induced DSBs are not significantly different in heterochromatin relative to euchromatin (69). In addition, proteins involved in DNA damage repair (9,70,71) are recruited to and form foci at similar rates in euchromatin and heterochromatin, implying that the chromatin state does not greatly affect access of some DSB signaling/repair proteins. Similarly, phosphorylation of H2AX and recruitment of XRCC1 is rapid in response to lesions induced in heterochromatin (9). Our preliminary finding that the recruitment and loss of Ku80-EGFP and DNA-PKcs-YFP to either NIR microbeam or USX-induced DSBs is unaffected by HDAC inhibition, suggests the affects by these inhibitors on the packaging of the DNA within the nucleus does not influence greatly accessibility of NHEJ repair proteins to damaged DNA (Supplementary Figure S7). Taken together, these latter findings are consistent with DSB complexity predominantly dictating the rate of NHEJ repair and the choice of NHEJ proteins recruited, although chromatin compaction may also play some role in other repair pathways.

**Figure 7.**
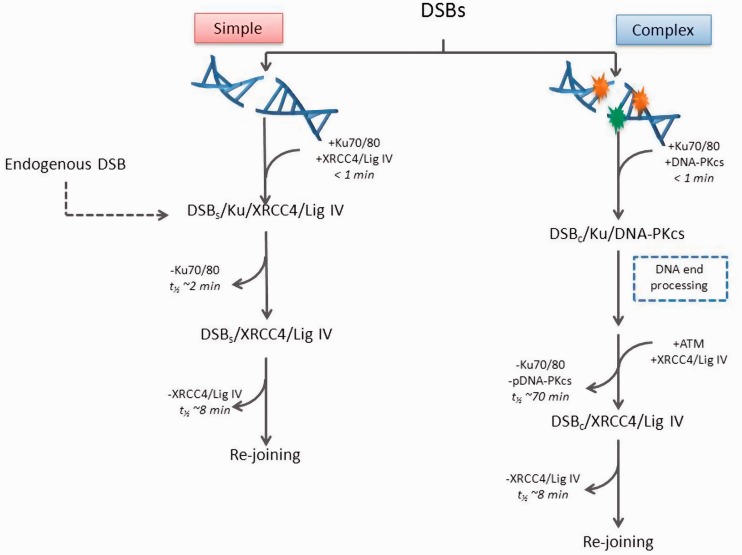
Schematic diagram illustrating the repair of simple DSBs and complex DSBs via NHEJ sub-pathways.

The following scheme is proposed for the repair pathways utilized by DSBs with varying degrees of chemical complexity (Figure 7). The repair of DSBs proceeds by two distinct sub-pathways of NHEJ. Simple DSBs are repaired rapidly and involve Ku70/80 and XRCC4/Ligase IV/XLF. The repair of complex DSBs requires recruitment of DNA-PKcs by Ku70/80 to form the DNA-PK complex which then recruits proteins involved in DNA end processing to remove lesions formed within close proximity to the DSB ends (Figure 7). Once the ‘clean-up’ of DSB ends has been completed, DNA-PKcs is phosphorylated by ATM and/or autophosphorylated to facilitate release of not only DNA-PKcs but also Ku80. Immediately prior to their release, XRCC4 is recruited to ensure ligation of the DSB. We propose that the complex DSBs are finally ligated by XRCC4/Ligase IV/XLF at a similar rate to that for simple DSBs. Therefore, the repair of DSBs is highly regulated with pathway choice and kinetics of repair dependent on the complexity of the DSBs.

## SUPPLEMENTARY DATA

Supplementary Data are available at NAR Online: Supplementary Figures 1–7, Supplementary Methods and Supplementary Reference [72].

## FUNDING

Science and Technology Facilities Council Biomed Network [HNB3003]; Medical Research Council [89975]; US Department of Energy [DE-SC0002296]. Funding for open access charge: Medical Research Council.

*Conflict of interest statement*. None declared.

## Supplementary Material

Supplementary Data

## References

[1] Jenner TJ, deLara CM, O'Neill P, Stevens DL (1993). Induction and rejoining of DNA double-strand breaks in V79-4 mammalian cells following gamma- and alpha-irradiation. Int. J. Radiat. Biol..

[2] Riballo E, Kuhne M, Rief N, Doherty A, Smith GC, Recio MJ, Reis C, Dahm K, Fricke A, Krempler A (2004). A pathway of double-strand break rejoining dependent upon ATM, Artemis, and proteins locating to gamma-H2AX foci. Mol. Cell.

[3] Shibata A, Conrad S, Birraux J, Geuting V, Barton O, Ismail A, Kakarougkas A, Meek K, Taucher-Scholz G, Lobrich M (2011). Factors determining DNA double-strand break repair pathway choice in G2 phase. EMBO.

[4] Karlsson KH, Radulescu I, Rydberg B, Stenerlow B (2008). Repair of radiation-induced heat-labile sites is independent of DNA-PKcs, XRCC1 and PARP. Radiat. Res..

[5] Dobbs TA, Palmer P, Maniou Z, Lomax ME, O'Neill P (2008). Interplay of two major repair pathways in the processing of complex double-strand DNA breaks. DNA repair.

[6] Asaithamby A, Uematsu N, Chatterjee A, Story MD, Burma S, Chen DJ (2008). Repair of HZE-particle-induced DNA double-strand breaks in normal human fibroblasts. Radiat. Res..

[7] Botchway SW, Stevens DL, Hill MA, Jenner TJ, O'Neill P (1997). Induction and rejoining of DNA double-strand breaks in Chinese hamster V79-4 cells irradiated with characteristic aluminum K and copper L ultrasoft X rays. Radiat. Res..

[8] Jeggo PA, Lobrich M (2005). Artemis links ATM to double strand break rejoining. Cell cycle.

[9] Jakob B, Splinter J, Conrad S, Voss KO, Zink D, Durante M, Lobrich M, Taucher-Scholz G (2011). DNA double-strand breaks in heterochromatin elicit fast repair protein recruitment, histone H2AX phosphorylation and relocation to euchromatin. Nucleic Acids Res..

[10] Rothkamm K, Lobrich M (2003). Evidence for a lack of DNA double-strand break repair in human cells exposed to very low x-ray doses. Proc. Natl Acad. Sci. USA.

[11] Goodhead DT (1994). Initial events in the cellular effects of ionizing radiations: clustered damage in DNA. Int. J. Radiat. Biol..

[12] Datta K, Jaruga P, Dizdaroglu M, Neumann RD, Winters TA (2006). Molecular analysis of base damage clustering associated with a site-specific radiation-induced DNA double-strand break. Radiat. Res..

[13] Datta K, Neumann RD, Winters TA (2005). Characterization of complex apurinic/apyrimidinic-site clustering associated with an authentic site-specific radiation-induced DNA double-strand break. Proc. Natl Acad. Sci. USA.

[14] Datta K, Neumann RD, Winters TA (2005). Characterization of a complex 125I-induced DNA double-strand break: implications for repair. Int. J. Radiat. Biol..

[15] Datta K, Weinfeld M, Neumann RD, Winters TA (2007). Determination and analysis of site-specific 125I decay-induced DNA double-strand break end-group structures. Radiat. Res..

[16] Henner WD, Grunberg SM, Haseltine WA (1982). Sites and structure of gamma radiation-induced DNA strand breaks. J. Biol. Chem..

[17] Henner WD, Rodriguez LO, Hecht SM, Haseltine WA (1983). gamma Ray induced deoxyribonucleic acid strand breaks. 3′ Glycolate termini. J. Biol. Chem..

[18] Feingold JM, Masch J, Maio J, Mendez F, Bases R (1988). Base sequence damage in DNA from X-irradiated monkey CV-1 cells. Int. J. Radiat. Biol. Relat. Stud. Phys. Chem. Med..

[19] van Gent DC, Hoeijmakers JH, Kanaar R (2001). Chromosomal stability and the DNA double-stranded break connection. Nat. Rev. Genet..

[20] Lieber MR (2010). The mechanism of double-strand DNA break repair by the nonhomologous DNA end-joining pathway. Annu. Rev. Biochem..

[21] Yaneva M, Kowalewski T, Lieber MR (1997). Interaction of DNA-dependent protein kinase with DNA and with Ku: biochemical and atomic-force microscopy studies. EMBO J..

[22] Yoo S, Dynan WS (1999). Geometry of a complex formed by double strand break repair proteins at a single DNA end: recruitment of DNA-PKcs induces inward translocation of Ku protein. Nucleic Acids Res..

[23] Bekker-Jensen S, Lukas C, Kitagawa R, Melander F, Kastan MB, Bartek J, Lukas J (2006). Spatial organization of the mammalian genome surveillance machinery in response to DNA strand breaks. J. Cell Biol..

[24] Uematsu N, Weterings E, Yano K, Morotomi-Yano K, Jakob B, Taucher-Scholz G, Mari PO, van Gent DC, Chen BP, Chen DJ (2007). Autophosphorylation of DNA-PKCS regulates its dynamics at DNA double-strand breaks. J. Cell Biol..

[25] Mari PO, Florea BI, Persengiev SP, Verkaik NS, Bruggenwirth HT, Modesti M, Giglia-Mari G, Bezstarosti K, Demmers JA, Luider TM (2006). Dynamic assembly of end-joining complexes requires interaction between Ku70/80 and XRCC4. Proc. Natl Acad. Sci. USA.

[26] Drouet J, Frit P, Delteil C, de Villartay JP, Salles B, Calsou P (2006). Interplay between Ku, Artemis, and the DNA-dependent protein kinase catalytic subunit at DNA ends. J. Biol. Chem..

[27] Budman J, Chu G (2005). Processing of DNA for nonhomologous end-joining by cell-free extract. EMBO J..

[28] Chappell C, Hanakahi LA, Karimi-Busheri F, Weinfeld M, West SC (2002). Involvement of human polynucleotide kinase in double-strand break repair by non-homologous end joining. EMBO J..

[29] Karimi-Busheri F, Rasouli-Nia A, Allalunis-Turner J, Weinfeld M (2007). Human polynucleotide kinase participates in repair of DNA double-strand breaks by nonhomologous end joining but not homologous recombination. Cancer Res..

[30] Macrae CJ, McCulloch RD, Ylanko J, Durocher D, Koch CA (2008). APLF (C2orf13) facilitates nonhomologous end-joining and undergoes ATM-dependent hyperphosphorylation following ionizing radiation. DNA Repair.

[31] Callebaut I, Malivert L, Fischer A, Mornon JP, Revy P, de Villartay JP (2006). Cernunnos interacts with the XRCC4 x DNA-ligase IV complex and is homologous to the yeast nonhomologous end-joining factor Nej1. J. Biol. Chem..

[32] Ahnesorg P, Smith P, Jackson SP (2006). XLF interacts with the XRCC4-DNA ligase IV complex to promote DNA nonhomologous end-joining. Cell.

[33] Kuhne M, Riballo E, Rief N, Rothkamm K, Jeggo PA, Lobrich M (2004). A double-strand break repair defect in ATM-deficient cells contributes to radiosensitivity. Cancer Res..

[34] Lobrich M, Jeggo PA (2005). Harmonising the response to DSBs: a new string in the ATM bow. DNA Repair.

[35] Datta K, Purkayastha S, Neumann RD, Pastwa E, Winters TA (2011). Base damage immediately upstream from double-strand break ends is a more severe impediment to nonhomologous end joining than blocked 3′-termini. Radiat. Res..

[36] Yano K, Morotomi-Yano K, Wang SY, Uematsu N, Lee KJ, Asaithamby A, Weterings E, Chen DJ (2008). Ku recruits XLF to DNA double-strand breaks. EMBO Rep..

[37] Kim JS, Krasieva TB, Kurumizaka H, Chen DJ, Taylor AM, Yokomori K (2005). Independent and sequential recruitment of NHEJ and HR factors to DNA damage sites in mammalian cells. J. Cell Biol..

[38] Hammel M, Yu Y, Mahaney BL, Cai B, Ye R, Phipps BM, Rambo RP, Hura GL, Pelikan M, So S (2010). Ku and DNA-dependent protein kinase dynamic conformations and assembly regulate DNA binding and the initial non-homologous end joining complex. J. Biol. Chem..

[39] Shao Z, Davis AJ, Fattah KR, So S, Sun J, Lee KJ, Harrison L, Yang J, Chen DJ (2012). Persistently bound Ku at DNA ends attenuates DNA end resection and homologous recombination. DNA Repair.

[40] Chen BP, Uematsu N, Kobayashi J, Lerenthal Y, Krempler A, Yajima H, Lobrich M, Shiloh Y, Chen DJ (2007). Ataxia telangiectasia mutated (ATM) is essential for DNA-PKcs phosphorylations at the Thr-2609 cluster upon DNA double strand break. J. Biol. Chem..

[41] Nikjoo H, O'Neill P, Terrissol M, Goodhead DT (1999). Quantitative modelling of DNA damage using Monte Carlo track structure method. Radiat. Environ. Biophys..

[42] Nikjoo H, Uehara S, Wilson WE, Hoshi M, Goodhead DT (1998). Track structure in radiation biology: theory and applications. Int. J. Radiat. Biol..

[43] Nikjoo H, Goodhead DT (1991). Track structure analysis illustrating the prominent role of low-energy electrons in radiobiological effects of low-LET radiations. Phys. Med. Biol..

[44] de Lara CM, Hill MA, Jenner TJ, Papworth D, O'Neill P (2001). Dependence of the yield of DNA double-strand breaks in Chinese hamster V79-4 cells on the photon energy of ultrasoft X rays. Radiat. Res..

[45] Harper JV, Reynolds P, Leatherbarrow EL, Botchway SW, Parker AW, O'Neill P (2008). Induction of persistent double strand breaks following multiphoton irradiation of cycling and G1-arrested mammalian cells-replication-induced double strand breaks. Photochem. Photobiol..

[46] Bekker-Jensen S, Lukas C, Melander F, Bartek J, Lukas J (2005). Dynamic assembly and sustained retention of 53BP1 at the sites of DNA damage are controlled by Mdc1/NFBD1. J. Cell. Biol..

[47] Botchway SW, Reynolds P, Parker AW, O'Neill P (2012). Laser-induced radiation microbeam technology and simultaneous real-time fluorescence imaging in live cells. Methods Enzymol..

[48] Hickson I, Zhao Y, Richardson CJ, Green SJ, Martin NM, Orr AI, Reaper PM, Jackson SP, Curtin NJ, Smith GC (2004). Identification and characterization of a novel and specific inhibitor of the ataxia-telangiectasia mutated kinase ATM. Cancer Res..

[49] Loh VM, Cockcroft XL, Dillon KJ, Dixon L, Drzewiecki J, Eversley PJ, Gomez S, Hoare J, Kerrigan F, Matthews IT (2005). Phthalazinones. Part 1: the design and synthesis of a novel series of potent inhibitors of poly(ADP-ribose)polymerase. Bioorg. Med. Chem. Lett..

[50] Mitchell J, Smith GC, Curtin NJ (2009). Poly(ADP-Ribose) polymerase-1 and DNA-dependent protein kinase have equivalent roles in double strand break repair following ionizing radiation. Int. J. Radiat. Oncol. Biol. Phys..

[51] Miller KM, Tjeertes JV, Coates J, Legube G, Polo SE, Britton S, Jackson SP (2010). Human HDAC1 and HDAC2 function in the DNA-damage response to promote DNA nonhomologous end-joining. Nat. Struct. Mol. Biol..

[52] Rathmell WK, Chu G (1994). Involvement of the Ku autoantigen in the cellular response to DNA double-strand breaks. Proc. Natl Acad. Sci. USA.

[53] Chan DW, Lees-Miller SP (1996). The DNA-dependent protein kinase is inactivated by autophosphorylation of the catalytic subunit. J. Biol. Chem..

[54] Errami A, Smider V, Rathmell WK, He DM, Hendrickson EA, Zdzienicka MZ, Chu G (1996). Ku86 defines the genetic defect and restores X-ray resistance and V(D)J recombination to complementation group 5 hamster cell mutants. Mol. Cell. Biol..

[55] Hill MA, Stevens DL, Kadhim M, Blake-James M, Mill AJ, Goodhead DT (2006). Experimental techniques for studying bystander effects in vitro by high and low-LET ionising radiation. Radiat. Prot. Dosim..

[56] Gulston M, de Lara C, Jenner T, Davis E, O'Neill P (2004). Processing of clustered DNA damage generates additional double-strand breaks in mammalian cells post-irradiation. Nucleic Acids Res..

[57] Audebert M, Salles B, Calsou P (2004). Involvement of poly(ADP-ribose) polymerase-1 and XRCC1/DNA ligase III in an alternative route for DNA double-strand breaks rejoining. J. Biol. Chem..

[58] Wang H, Perrault AR, Takeda Y, Qin W, Iliakis G (2003). Biochemical evidence for Ku-independent backup pathways of NHEJ. Nucleic Acids Res..

[59] Wang M, Wu W, Wu W, Rosidi B, Zhang L, Wang H, Iliakis G (2006). PARP-1 and Ku compete for repair of DNA double strand breaks by distinct NHEJ pathways. Nucleic Acids Res..

[60] Kysela B, Doherty AJ, Chovanec M, Stiff T, Ameer-Beg SM, Vojnovic B, Girard PM, Jeggo PA (2003). Ku stimulation of DNA ligase IV-dependent ligation requires inward movement along the DNA molecule. J. Biol. Chem..

[61] Pang D, Yoo S, Dynan WS, Jung M, Dritschilo A (1997). Ku proteins join DNA fragments as shown by atomic force microscopy. Cancer Res..

[62] DiBiase SJ, Zeng ZC, Chen R, Hyslop T, Curran WJ, Iliakis G (2000). DNA-dependent protein kinase stimulates an independently active, nonhomologous, end-joining apparatus. Cancer Res..

[63] Anderson JA, Harper JV, Cucinotta FA, O'Neill P (2010). Participation of DNA-PKcs in DSB repair after exposure to high- and low-LET radiation. Radiat. Res..

[64] Darroudi F, Wiegant W, Meijers M, Friedl AA, van der Burg M, Fomina J, van Dongen JJ, van Gent DC, Zdzienicka MZ (2007). Role of Artemis in DSB repair and guarding chromosomal stability following exposure to ionizing radiation at different stages of cell cycle. Mutat. Res..

[65] Goodarzi AA, Yu Y, Riballo E, Douglas P, Walker SA, Ye R, Harer C, Marchetti C, Morrice N, Jeggo PA (2006). DNA-PK autophosphorylation facilitates Artemis endonuclease activity. EMBO J..

[66] Bennardo N, Stark JM (2010). ATM limits incorrect end utilization during non-homologous end joining of multiple chromosome breaks. PLoS Genet..

[67] Goodarzi AA, Noon AT, Deckbar D, Ziv Y, Shiloh Y, Lobrich M, Jeggo PA (2008). ATM signaling facilitates repair of DNA double-strand breaks associated with heterochromatin. Mol. Cell.

[68] Goodarzi AA, Kurka T, Jeggo PA (2011). KAP-1 phosphorylation regulates CHD3 nucleosome remodeling during the DNA double-strand break response. Nat. Struct. Mol. Biol..

[69] Rief N, Lobrich M (2002). Efficient rejoining of radiation-induced DNA double-strand breaks in centromeric DNA of human cells. J. Biol. Chem..

[70] Chiolo I, Minoda A, Colmenares SU, Polyzos A, Costes SV, Karpen GH (2011). Double-strand breaks in heterochromatin move outside of a dynamic HP1a domain to complete recombinational repair. Cell.

[71] Asaithamby A, Hu B, Chen DJ (2011). Unrepaired clustered DNA lesions induce chromosome breakage in human cells. Proc. Natl Acad. Sci. USA.

[72] Goodhead DT, Thacker J, Cox R (1979). Effectiveness of 0.3 keV carbon ultrasoft X-rays for the inactivation and mutation of cultured mammalian cells. Int. J. Radiat. Biol. Relat. Stud. Phys. Chem. Med..

