# Supplementary material for: The dynamics of Ku70/80 and DNA-PKcs at DSBs induced by ionizing radiation is dependent on the complexity of damage
Source: Nucleic Acids Res. 2012 Sep 24;40(21):10821–31. doi: 10.1093/nar/gks879 (PMC3510491; doi:10.1093/nar/gks879)
Supplement: Supplementary Data [file supp_40_21_10821__index.html]

The dynamics of Ku70/80 and DNA-PKcs at DSBs induced by ionizing radiation is dependent on the complexity of damage — The dynamics of Ku70/80 and DNA-PKcs at DSBs induced by ionizing radiation is dependent on the complexity of damage — Supplementary Data 

# The dynamics of Ku70/80 and DNA-PKcs at DSBs induced by ionizing radiation is dependent on the complexity of damage

## Supplementary Data

files

**Files in this Data Supplement:**

- Supplementary Data - docx file
