# Supplementary material for: The dynamics of Ku70/80 and DNA-PKcs at DSBs induced by ionizing radiation is dependent on the complexity of damage
Source: Nucleic Acids Res. 2012 Sep 24;40(21):10821–31. doi: 10.1093/nar/gks879 (PMC3510491; doi:10.1093/nar/gks879)
Supplement: Supplementary Data [file supp_gks879_nar-02040-d-2012-File009.docx]

**Supplementary information**

**Materials and Methods**

**Stable expression of XRCC4-GFP in XR1 cells.** XR1 cells were plated at 2.0 x 10^5^ cells per 60 mm dish 24 h prior to transfection in 5 ml of medium. Cells were transfected with GFP-tagged ORF clone of XRCC4 (Insight Biotechnology, UK) using SuperFect^®^ (Qiagen, UK) according to the manufacturers protocol. The cells were incubated for 24 h under normal culture conditions. The cells were split 1:10 into 60 mm dishes containing complete growth medium with 1 mg/ml of G418 (PAA UK, the concentration was previously determined by conducting a G418 killing curve) until all the cells in the control dish had died. The transfected cells were serial diluted in a 96 well plate (growth media containing 0.4 mg/ml G418) to obtain a XRCC4-GFP cell population originating from a single cell. The fluorescence intensity levels were then determined using confocal microscopy to select stable clones containing GFP tagged XRCC4. Protein expression levels were then determined by western blot analysis of GFP-tagged XRCC4 compared to wild type CHO cells containing endogenous XRCC4 (Fig. S1).

**Aluminium K-shell ultrasoft X-ray set-up.** These USX, which are highly attenuated and so are easily shielded, interacting within the cell via the photoelectric effect to give two electrons with a combined range of around 70 nm with negligible scattered USX and are therefore ideal for partial shielding experiments. Partial shielding was achieved by irradiating through a mask in direct contact with the dish base. The mask consisted of a patterned 1 μm layer of gold on a 0.5 μm silicon nitride membrane supported by silicon with a 0.9 μm Mylar protective layer.

The dish and mask were positioned directly above the USX source using a brass holder that was thermostatically controlled by a pumped water circulator. Dosimetry was performed using an air filled ion chamber with an internal diameter of 0.8 cm, volume of 0.1 cm^3^ and a 0.262 mg cm^-2^ aluminium foil window which was positioned parallel to and 1 mm behind an empty Mylar dish. The ionization current through the gold mask was measured using a Keithley 6517 electrometer and the mean dose rate to the cell was calculated in a similar manner to Goodhead *et al* (1) based on a mean cell thickness of 5 μm. The contribution to the dose from transmission through the gold is approximately 7%.

Al_K_ characteristic USX (1.49 keV) were produced using a cold cathode discharge tube (5 kV, 5 mA) with an aluminum foil transmission target (1.27 mg cm^-2^) with bremsstrahlung contributing to <10% of the dose (33,45). The X-rays travel 5.4 cm through a flight tube, which is continuously flushed with helium (1500 cm^3^ min^-1^) at atmospheric pressure before irradiating the cells in glass wall dishes with 0.9 μm Mylar bases through a 1 µm x 9 µm gold grid (Fig S2).

**NIR laser microbeam set-up.** The NIR laser microbeam was generated using a Ti:sapphire (Mira 900, Coherent Lasers, UK) laser system with a repetition rate of 76 MHz and a pulse width of 180 fs. The laser beam was expanded and reflected into the back of an inverted microscope (Nikon Instruments Europe B. V., UK). The microscope was fitted with a computer controlled microscope stage for automated movement (Märzhäuser Wetzlar GmbH & Co., Germany). The laser microbeam is typically focused to ~1 µm spot size. To calculate the potency of the ultra-short pulse laser to induce DNA damage, the peak power of the laser pulse must be considered, not the average power, which is the light energy in 1 s. Thus, 1 mW average power means each laser pulse gives a power/repletion rate of 1 x 10^-3^ mW/76 x 10^-6^ s^-1^ equal to 1.3 x 10^-11^ J. This gives a peak power of 73 W (1.3 x 10^-11^ J/180 x 10^-15^ s), or assuming a 1 μm spot, the laser irradiance is 9.3 GW cm^-2^. In reality, the pulse width is lengthened by the diffracting optical lens system to ~500 fs thus the laser irradiance is ~3.4 GWcm^-2^.

**Flow cytometric analysis.** Cells were incubated for 30 min at 37°C with 10 µM BrdU prior to fixation. Cells were harvested by trypsinization and centrifuged. The supernatant was aspirated and the cell pellet was washed in PBS followed by centrifugation. The cell pellet was re-suspended in ice cold 70% ethanol for a minimum of 30 min. The cells were centrifuged and treated with 1% hydrochloric acid at 37°C for 10 min. PBS was added before centrifugation and 100 µl of anti-BrdU solution was added (5% Tween, 1% FCS and 5 mg/ml anti-BrdU antibody [BD Biosciences] in PBS) at room temperature for 1 h. The cells were centrifuged before the addition of secondary antibody solution (5% Tween, 1% FCS and 50 µg/ml of FITC anti-mouse antibody in PBS) at room temperature for 30 min. Cells were stained in 1 ml staining solution containing 50 µg/ml propidium iodide and 20 mg/ml of RNase A in PBS. Cells were analyzed using a Becton Dickinson FACS Sort.

**Western blot analysis of protein expression.** Cells were plated in T25 flasks at 0.5 x 10^6^ cells per flask for 24 h. The protein was extracted using cell lysis buffer according to the manufacturers’ instructions (Cell Signalling, UK). The proteins were separated by SDS-PFGE using a 12% gel before transfer to a nitrocellulose membrane using an iBlot dry blotting system (Invitrogen, UK). The membranes were incubated with primary antibody (XRCC4 (C20) anti-goat, Santa Cruz, USA; β-actin anti-mouse, Abcam, UK) overnight followed by incubation with secondary antibody (donkey anti-goat IR dye 680T or goat anti-mouse IR dye 680, LiCOR Biosciences, UK) for 1 h at room temperature. The proteins were then visualized using an Odyssey imager (LI-COR Biosciences, UK).

**Gamma radiations and immunofluorescence staining of γ-H2AX.** For gamma radiations cells were placed on ice 10 min prior to irradiation. Cells were irradiated at room temperature in cell culture medium with 1 Gy ^137^Cs γ-rays (dose rate 1.6 Gy/min^-1^). The culture medium was then replaced with medium pre-warmed to 37°C and cells were incubated at 37°C with 5% CO_2_ humidified air for the stated repair times before fixation in 3% paraformaldehyde for a minimum of 30 min. Following fixation, cells were washed in PBS and permeabilised in 1% triton-X-100 in PBS for 10 min. Cells were washed and blocked in 1% fish skin gelatin, 1% bovine serum albumin in PBS for 1 h at room temperature. The cells were then incubated with mouse anti-histone 2A.X phosphorylated on ser139 (Abcam, UK) antibody at 1:300 dilution for 1 h at room temperature. Cells were then washed and incubated with anti-mouse FITC (V79-4 cells) or Cy3 (fluorescently tagged cells) (Stratech Scientific, UK) antibody at 1:100 dilution for 1 h at room temperature. Cells were washed in PBS and mounted in Vectashield^®^ anti-fading medium. Cells were imaged using confocal microscopy (Biorad Radiance 2000, Carl Zeiss Ltd UK) equipped with 405 nm (DAPI) and 488 nm (FITC) 535 nm (Cy3) lasers. The images were analyzed using ImageJ^®^ software.

**Reference**

1. Goodhead, D.T, Thacker J. and Cox R. (1979) [Effectiveness of 0.3 keV carbon ultrasoft X-rays for the inactivation and mutation of cultured mammalian cells.](http://www.ncbi.nlm.nih.gov/pubmed/315385) *Int J Radiat Biol Relat Stud Phys Chem Med,* **36**, 101-14.


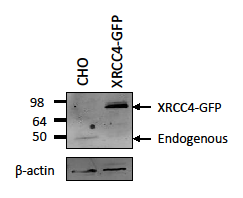


**S1:** Western blot analysis of the expression level of XRCC4-GFP in XR1 cells complemented with the XRCC4-GFP compared with endogenous levels in wild type CHO cells.

**S2:** a) Schematic representation of the micro-irradiation of cells with 1 μm wide stripes of ultrasoft x-rays using a 1 μm thick gold mask deposited in 9 μm wide stripes separated by 1 μm. b) Image of HD810 Gafchromic film irradiated in the position of the cells, demonstrating that the pattern of transmission of x-rays through the mask and the pattern of tracks of γH2AX in cells is as shown in Figure S5.

(b)

(a)

**S3:** (a) Following USX irradiation of Ku80-EGFP tagged cells in medium at 7°C, the time dependence of the increase in temperature of the medium was determined after addition of pre-warmed (37°C) cell medium and subsequent incubation at 37°C. (b) Real time recruitment and loss of fluorescence intensity of Ku80-EGFP on time following USX irradiation with 27 Gy at 37°C or 7°C. The graph represents the mean of 3 independent experiments ± SEM The kinetic analysis to obtain the best fit to the experimental data are shown as solid and dotted lines and described in the Material and Methods. The t_½_ value decreases from 11.5 ±5 to 7.1 ±3 min on increasing the temperature from 7°C to 37°C. Many of the simple DSBs could have been repaired during the irradiation time of 10 min at 37°C so that the kinetics of the persisting fluorescence intensity (~15%) will become more prominent in the kinetic analysis as seen from the fit to the data in S3b.

(a)

(b)

**S4:** Effects of 250 nM PARP inhibitor on real time recruitment and loss of fluorescence intensity of Ku80-EGFP. (a) Real time recruitment and loss of Ku80-EGFP following NIR microbeam irradiation with 730 nm photons (at a power of 10 mW using x60 objective) and at 37°C in the presence of PARP inhibitor. (b) Real time recruitment and loss of Ku80-EGFP following USX irradiation with 27 Gy at 7°C in the presence of PARP inhibitor. The graph represents the mean of 3 independent experiments ± SEM with the solid (control) and dotted (inhibitor) lines showing the fit of the exponential decays to the data points as described in the Materials and Methods.

(b)

(a)

**S5:** (a) Dose dependency of the fluorescence intensity of Ku80-EGFP and DNA-PKcs-YFP in the respective cells following USX irradiation of cycling cells at 7^o^C. Following USX irradiations cells were incubated at 37^o^C for 5 min prior to fixing and co-stained for γ-H2AX formation. As can be seen the foci tracks of Ku80-EGFP and DNA-PKcs-YFP co-localize with those for γH2AX, although the width of the track is greater for γH2AX than the other two proteins. The γH2AX signal is amplified as phosphorylation of H2AX is known to spread over hundreds of Mbp around the DSB, whereas few Ku and DNA-PKcs molecules are recruited and remain close to the actual DSB, thereby resulting in the differences seen in the definition of the foci tracks. (b) Time course of the loss of γ-H2AX foci at sites of DNA damage induced in cycling Ku80-EGFP tagged cells () and DNA-PKcs-YFP tagged cells (○) following 1 Gy γ-radiation. The cells were fixed at the stated repair times and the background foci were subtracted from each time point. The graphs represent the mean of 3 independent experiments ± SEM.

**S6:** Effects of 10 µM ATM kinase inhibitor of ATM kinase activity on the real time recruitment and loss of fluorescence intensity of Ku80-EGFP following USX irradiation of Ku80-EGFP tagged cells with 27 Gy at 37°C pre- and post-irradiation. The graph represents the mean of 3 independent experiments ± SEM with the solid (control) and dotted (inhibitor) lines showing the fit of the exponential decays to the data points as described in Materials and Methods, time zero refers to the start of the irradiation with USX.

(d)

(c)

(b)

(a)

(e)

**S7:** Effects of 5 mM sodium butyrate (NaB) and 1.3 µM trichostatin A (TSA) on the real time recruitment and loss of fluorescence intensity of DNA repair proteins. (a) and (b) Ku80-EGFP following NIR microbeam irradiation with 730 nm photons (at a power of 10 mW using x60 objective) at 37°C. (c) Ku80-EGFP following USX irradiation at 27 Gy. (d) and (e) DNA-PKcs-YFP following USX irradiation at 137 Gy at 7°C. The graph represents the mean of 3 independent experiments ± SEM with the solid (control) and dotted (inhibitor) lines showing the fit of the exponential decays to the data points as described in Materials and Methods.
